# Recombinant Adeno-Associated Virus-Mediated Expression of Methamphetamine Antibody Attenuates Methamphetamine-Induced Hyperactivity in Mice

**DOI:** 10.1038/srep46301

**Published:** 2017-04-07

**Authors:** Yun-Hsiang Chen, Kuo-Jen Wu, Kuang-Lun Wu, Kun-Lieh Wu, Ho-Min Tsai, Mao-Liang Chen, Yi-Wei Chen, Wei Hsieh, Chun-Ming Lin, Yun Wang

**Affiliations:** 1 Department of Life Science, Fu-Jen Catholic University, New Taipei City, Taiwan; 2 Center for Neuropsychiatric Research, National Health Research Institutes, Zhunan, Taiwan; 3 Department of Electrical Engineering of I-Shou University, Kaohsiung, Taiwan; 4 Department of Research, Taipei Tzu Chi Hospital, Buddhist Tzu Chi Medical Foundation, New Taipei City, Taiwan; 5 Department of Microbiology, Soochow University, Taipei, Taiwan

## Abstract

Methamphetamine (Meth) is one of the most frequently abused drugs worldwide. Recent studies have indicated that antibodies with high affinity for Meth reduce its pharmacological effects. The purpose of this study was to develop a technique for virus-based passive immunization against Meth effects. We generated a recombinant adeno-associated virus serotype-8 vector (AAV-MethAb) carrying the gene for a Meth-specific monoclonal antibody (MethAb). Infection of 293 cells with AAV-MethAb resulted in the expression and secretion of antibodies which bind to Meth. The viral vector was then examined in adult ICR mice. Systemic administration of AAV-MethAb resulted in long-term expression of MethAb in the serum for up to 29 weeks. Serum collected from the animals receiving AAV-MethAb retained a high specificity for (+)-Meth. Animals were challenged with Meth five weeks after viral injection. Meth levels in the brain and serum were reduced while Meth-induced locomotor activity was significantly attenuated. In conclusion, AAV-MethAb administration effectively depletes Meth from brain and serum while reducing the behavioral response to Meth, and thus is a potential therapeutic approach for Meth abuse.

Methamphetamine (Meth) abuse leads to cognitive dysfunction, neurodegeneration, infections, and numerous complications^1^,^2^,^3^, and has become a public health and social concern around the world. Currently, there is no effective pharmacological therapy for Meth abuse in patients^4^,^5^. Selective antibodies against Meth have been examined for the treatment of Meth addiction through active or passive immunization^6^,^7^, an approach that was initially developed for the treatment of snake venom or digoxin^8^. In active immunization, Meth-like small molecules, linked to immunogenic carriers, were used to stimulate the production of Meth antibodies in hosts^7^,^9^,^10^. For passive immunization, hosts were infused intravenously with specific Meth antibodies derived from vaccinated animals or the synthetic antibody libraries^11^,^12^,^13^. These high-affinity Meth antibodies capture the Meth molecule in the circulatory system, reduce its access to the activation sites in the brain, and attenuate Meth-mediated behavioral changes^6^,^11^,^14^,^15^.

The effectiveness of Meth antibodies is generally limited by several factors. For example, Meth is a weak immunogen; a regular and well-scheduled course of vaccination is required to maintain Meth antibody titer in the blood^9^,^16^. The antibodies induced by active immunization may cross-react with other molecules or endogenous ligands structurally similar to Meth. Furthermore, HIV or other immune-compromised conditions, commonly seen in drug addicts, may yield insufficient antibodies after active immunization^17^,^18^. These limitations may be partially overcome by passive immunization. However, repeated administration of purified Meth antibodies would generally be required to reduce Meth responses over an extended period of time, as would be necessary for chronic users. The high cost of passive immunization would also be a complicating factor likely to reduce compliance in Meth addicts.

We hereby propose an alternative approach to producing Meth antibodies in the host through adeno-associated virus (AAV) infection. A recombinant AAV serotype-8 vector (AAV-MethAb) carrying the Meth monoclonal antibody gene was used to express a monoclonal antibody specific for Meth *in vivo*. The AAV system is non-pathogenic, has low immunogenicity and has been considered a safe and ideal vector system for gene therapy^19^,^20^,^21^. For this paper, both the heavy-chain and light-chain genes of the Meth antibody were linked together by a self-cleavage 2A sequence and cloned in the same expression cassette. This approach was taken to obtain high expression levels of full-length antibodies and to overcome the AAV packaging limit (~5 kb). The 2A sequence, derived from the foot-and-mouth disease virus, disrupts the peptide bond formation at its C-terminus during translation, leading to co-expression of two proteins flanking the 2A sequence at similar levels from a single open reading frame^22^.

Our results demonstrate that systemic administration of AAV-MethAb induced long-term and stable expression of a Meth-specific monoclonal antibody (MethAb) in the blood. Animals receiving AAV-MethAb had a lower Meth level in brain and attenuated Meth-mediated locomotor activity after Meth administration. Our data suggest that AAV-mediated expression of Meth antibodies in the periphery could be developed as a treatment for the Meth abuse and intoxication.

## Results

### Expression of Meth antibodies in cultured 293 cells

**Figure 1:**
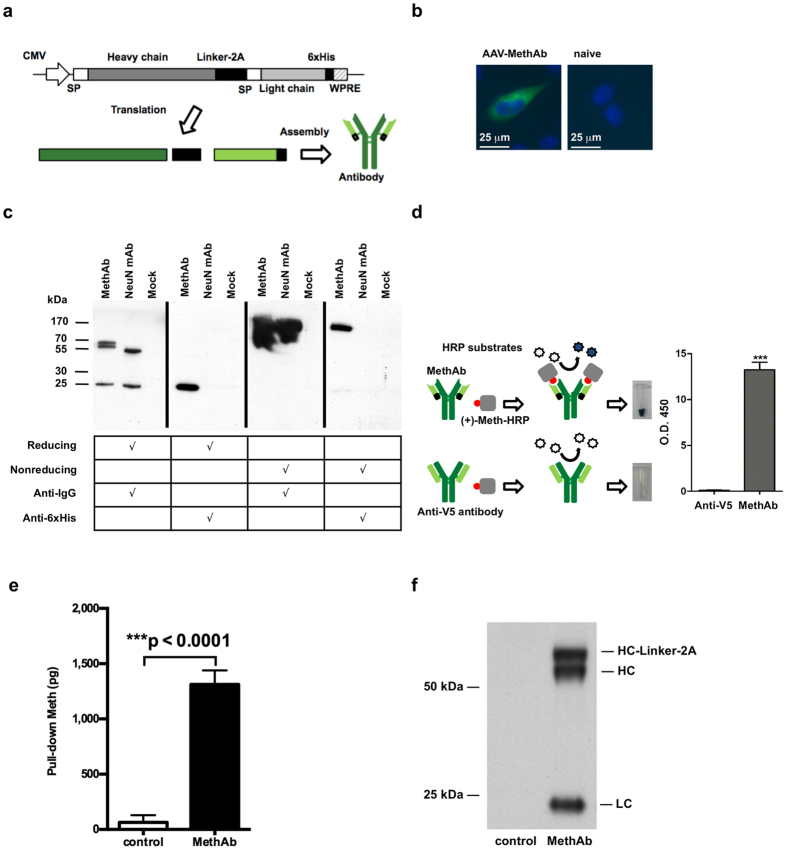
Characterization of the MethAb. (**a**) The genetic construct of the Meth-specific monoclonal antibody (MethAb). Both heavy-chain and light-chain genes of MethAb were linked by a 2A self-cleavage sequence and driven by the cytomegalovirus (CMV) promoter in the expression vector pAAV-MethAb. Linker = a sequence composed of a furin cleavage site (RKRR), a V5 epitope, and a spacer peptide (SGSG); SP = signal peptide; WPRE = woodchuck hepatitis B virus post-transcriptional regulatory element; 6 × His = 6 × histidine tag. (**b**) Mouse IgG (green) immunoreactivity was found in 293 cells pretreated with AAV-MethAb, but not in naïve cells. Cellular nuclei were labeled with DAPI (blue). Calibration = 25 μm. (**c**) A secreted full-length antibody was generated by 293 cells transduced with AAV-MethAb. Culture media were harvested from transduced cells and subjected to antibody purification by using protein-G conjugated magnetic beads. Purified samples were separated by SDS-PAGE under reducing or nonreducing conditions and probed with an anti-mouse-IgG polyclonal antibody or an anti-6 × His polyclonal antibody in Western blot analysis. Mock = samples were purified from the culture media of un-transduced 293 cells; NeuN = a commercially available mouse mAb recognizing the neuron-specific nuclear protein. (**d**) The antibody, generated by AAV-MethAb transduced 293 cells, can bind to Meth-HRP. Purified MethAb was subjected to a colorimetric assay to assess its binding activity to Meth molecules. The conversion of horseradish peroxidase (HRP) substrates to blue-colored substances was measured at 450 nm. ***p < 0.0001. Anti-V5 = a mouse mAb recognizing the V5 epitope derived from the simian parainfluenza virus type 5. (**e**) MethAb can pull down Meth. 293 cells were transfected with pAAV-MethAb or transfection reagent only (control). The secreted MethAb was purified by His-Mag Sepharose beads at 48 h post-transfection and then incubated with 150 μl of Meth (50 ng/ml) to pull down Meth molecules. After that, beads were collected by a magnet, and the supernatant was subjected to the quantification of Meth. The amount of pulled-down Meth was calculated. (**f**) The purified MethAb used for the precipitation of Meth was separated by SDS-PAGE under the reducing condition. The heavy chain (HC, HC-Linker-2A) and light chain (LC) of MethAb were detected by an anti-mouse-IgG goat polyclonal antibody in Western blot analysis.

The mouse monoclonal antibody clone 6H4 (MethAb), with high affinity (K_D_ = 11 nM) to Meth, was chosen as a model antibody. To facilitate antibody assembly and overcome the limitation of AAV packaging size, the heavy-chain and light-chain genes of MethAb were linked together by a 2A self-processing sequence and constructed as a single open reading frame flanked by AAV2 inverted repeats (Fig. 1a). The construct was used to generate the pseudo-serotype-8 viral vector, AAV-MethAb, by triple transfection. We first transduced 293 cells with AAV-MethAb at a multiplicity of infection (MOI) of 10^2^ viral genome copies per cell (VGC/cell). At 48 h post-transduction, cells were fixed and stained with a fluorescence-labeled anti-mouse IgG antibody. The expression of MethAb was located in the cytoplasmic compartment (Fig. 1b).

The antibodies secreted into cell culture media were purified by protein-G beads and examined in reducing or nonreducing conditions through Western blot analysis (Fig. 1c). In the reducing condition where the antibody’s disulfide bonds were broken, the heavy (~60 kDa) and light chains (~25 kDa) of MethAb were detected by an anti-mouse IgG antibody, whereas a single protein band of approximately 25 kDa was detected by an anti-6 × His antibody. These data suggest that the 2A self-processing sequence facilitated co-expression of both heavy and light chains of MethAb from a single open reading frame. An extra protein band (~65 kDa) was found near the predicted heavy chain of MethAb, probably due to an incomplete removal of the Linker-2A sequence by furin cleavage. In contrast, in the nonreducing condition where the antibody’s disulfide bonds were not broken, a single protein band of ~170 kDa, similar to the size of a dimerized full-length antibody, was detected by either anti-mouse IgG or anti-6 × His antibodies. These data suggest that AAV-MethAb infection increased expression of secreted full-length antibodies in a self-assembling soluble form in the cultured cells.

### Meth bound to the anti-Meth antibody

The antibody was purified from the cell culture media, and a viral-specific antibody (anti-V5) was used as a control. After addition of colorless HRP substrates, blue-colored products were generated only in the samples containing the purified MethAb, but not anti-V5 (Fig. 1d), suggesting that the secreted antibodies derived from AAV-MethAb transduced cells retained binding activity to (+)-Meth-HRP. The Meth binding activity was also confirmed by the assay showing direct pull-down of (+)-Meth molecules (Fig. 1e, 1f). Based on the pull-down assay, 1.2 μg of MethAb was shown to precipitate 1,248 ± 126 pg of (+)-Meth. MethAb is composed of 1,416 amino acids, and the molecular weight (MW) is estimated to be 155,760 Da; the MW of (+)-Meth is 150 Da. The binding capacity of MethAb was calculated using the formula described in Methods (Binding capacity of anti-Meth antibody) and estimated to be 1.1 ± 0.11 molecules of Meth per molecule of MethAb; (1,248/150)/(1.2 × 10^6^/155,760) = 1.1.

### Long-term expression of anti-Meth antibodies *in vivo*

**Figure 2:**
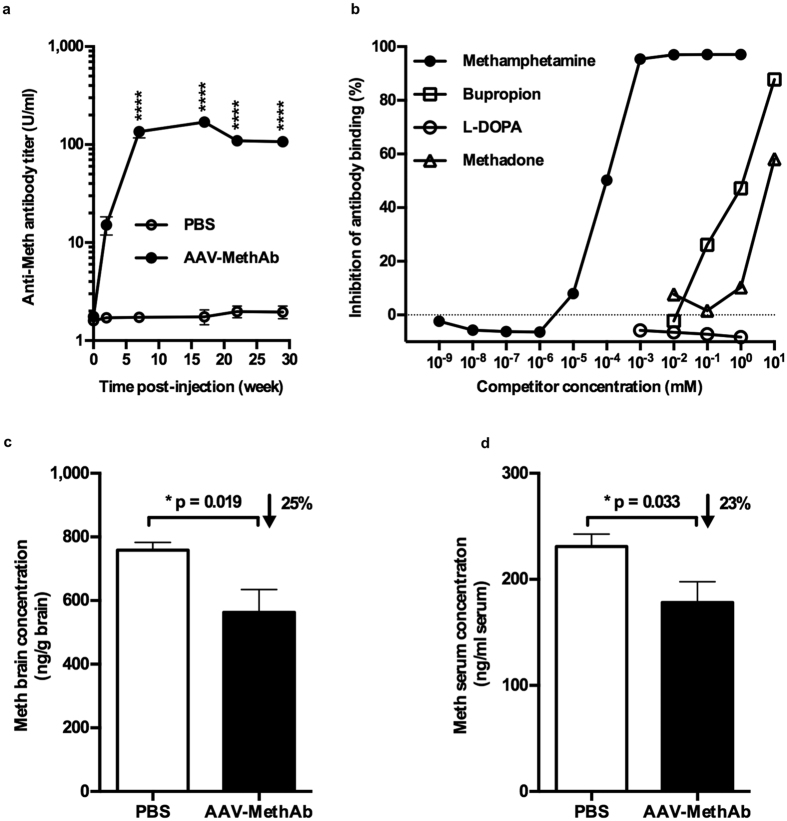
AAV-MethAb infection induced long-lasting expression of MethAb in serum and reduced Meth levels in the brain and serum. (**a**) Mice were injected with AAV-MethAb (10^9^ VGC/animal; n = 3, i.p.) or PBS (n = 4). Significant increases in MethAb expression in serum were found up to 29 weeks after AAV-MethAb injection (p < 0.0001, 2-way ANOVA; AAV-MethAb vs. PBS at 7, 17, 22, and 29 weeks post-injection). (**b**) Serum samples were pooled at 9 weeks after AAV-MethAb injection. Meth, but not other ligands, selectively competed with Meth-HRP for MethAb binding at a concentration of 10^−5^ mM. (**c**,**d**) At 15 weeks post-injection, all animals received Meth challenge (1 mg/kg, i.p.); brain and blood samples were collected 10 minutes later. The Meth concentrations in the brain and serum were significantly reduced (*p < 0.05, Student’s *t*-test) in AAV-MethAb injected mice.

Adult male ICR mice (8 weeks) received AAV-MethAb (n = 3, at a dose of 10^9^ VGC/animal, i.p.) or PBS (n = 4). Serum samples were collected before (0 weeks) and 2, 7, 17, 22, 29 weeks after virus injection (Fig. 2a). No anti-Meth antibody was found before the virus or PBS injection in week 0. In animals receiving AAV-MethAb, anti-Meth antibodies were first detected at 2 weeks, peaked (1.7 × 10^2^ U/ml) at 7 weeks, and maintained at steady state to the last evaluated time point at the 29th week. These data suggest that a single intraperitoneal administration of AAV-MethAb resulted in a persistent long-term expression of anti-Meth antibodies in the peripheral blood.

We further examined the specificity of anti-Meth antibodies *in vivo* (Fig. 2b). Mice (n = 10) were treated with AAV-MethAb (10^9^ VGC/animal, i.p.). Sera were collected at 9 weeks post-injection. Four molecules (bupropion, L-DOPA, methadone, and Meth) were used to compete with (+)-Meth-HRP for the binding to serum-derived anti-Meth antibodies. The IC50 for Meth (~10^−4^ mM) was much lower (>10,000-fold) than that for bupropion (~1 mM), L-DOPA (~10 mM), and methadone (>1 mM), suggesting that the antibodies derived from the AAV-MethAb infection were specific to (+)-Meth *in vivo*.

### Peripheral anti-Meth antibodies reduced Meth levels in the brain and serum

At 15 weeks after AAV-MethAb (10^9^ VGC/animal; i.p., n = 10) or PBS (n = 10) administration, mice were injected with Meth (1 mg/kg, i.p.). Animals were sacrificed 10 min later for the measurement of Meth levels in the brain and serum. Since the presence of MethAb might affect the accuracy of measuring unbound Meth in the periphery by ELISA, these serum samples were pretreated with protein-G beads to deplete antibodies. We found that Meth levels in the brain and serum were significantly reduced by 25% (p = 0.019) and 23% (p = 0.033), respectively, in mice receiving AAV-MethAb, compared to the non-infected mice (Fig. 2c, 2d). No significant difference in body weight (43.9 ± 4.7 g vs. 44.3 ± 4.8 g) or brain weight (0.53 ± 0.04 g vs. 0.51 ± 0.04 g) was observed between AAV-MethAb injected and PBS-injected mice.

### AAV-MethAb infection attenuated Meth-induced locomotor activity in mice

**Figure 3:**
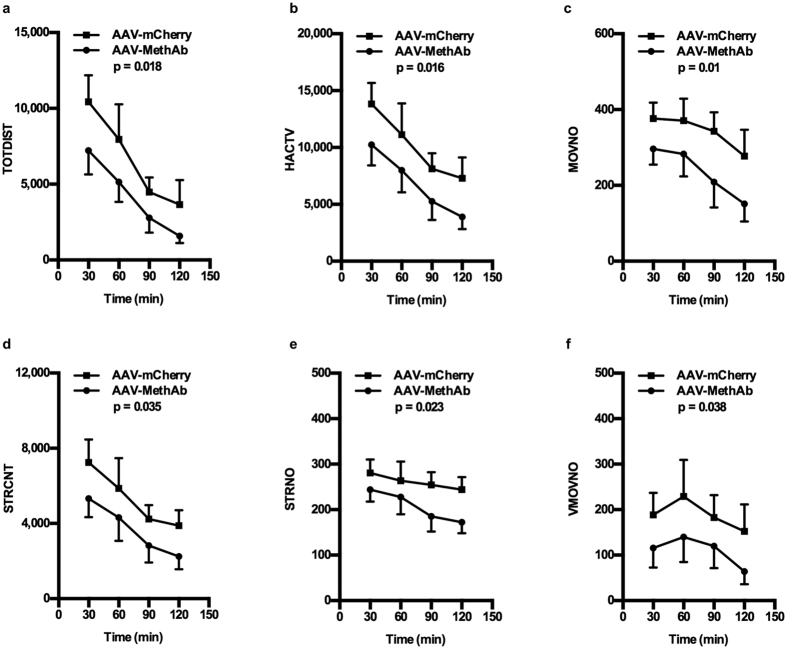
Pretreatment with AAV-MethAb attenuates Meth-induced locomotor activity in mice. Mice receiving AAV-MethAb (2.5 × 10^10^ VGC/animal; n = 8, i.p.) or AAV-mCherry (2.5 × 10^10^ VGC/animal; n = 6, i.p.) were challenged with Meth (1 mg/kg, i.p.) at five weeks post-infection, and behavioral measurements were performed in the activity chambers for 2 h. The effect of AAV-MethAb was assessed by the following measures (p values in parentheses are for the main effect of treatment, comparing AAV-MethAb to AAV-mCherry). None of the interaction effects (time vs. treatment) were statistically significant. (**a**) Total distance traveled (TOTDIST) (F_1,48_ = 5.993, p = 0.018); Time: F_3,48_ = 7.928, p < 0.001; Interaction: F_3,48_ = 0.119, p = 0.949. (**b**) Horizontal activity (HACTV) (F_1,48_ = 6.289, p = 0.016); Time: F_3,48_ = 5.027, p = 0.004; Interaction: F_3,48_ = 0.0154, p = 0.997. (**c**) Movement number (MOVNO) (F_1,48_ = 7.229, p = 0.01); Time: F_3,48_ = 1.975, p = 0.013; Interaction: F_3,48_ = 0.115, p = 0.951. (**d**) Stereotype count (STRCNT) (F_1,48_ = 4.708, p = 0.035); Time: F_3,48_ = 3.850, p = 0.015; Interaction: F_3,48_ = 0.02, p = 0.996. (**e**) Stereotype number (STRNO) (F_1,48_ = 5.49, p = 0.023); Time: F_3,48_ = 1.166, p = 0.332; Interaction: F_3,48_ = 0.192, p = 0.901. (**f**) Vertical movement number (VMOVNO) (F_1,48_ = 4.535, p = 0.038); Time: F_3,48_ = 0.772, p = 0.544; Interaction: F_3,48_ = 0.0297, p = 0.993.

A total of 14 mice were infected with AAV-MethAb (2.5 × 10^10^ VGC/animal; i.p., n = 8) or AAV-mCherry (2.5 × 10^10^ VGC/animal; i.p., n = 6). At five weeks post-infection, mice were injected with a dose of Meth (1 mg/kg, i.p.) and placed individually into activity chambers to record behavioral activity for 2 h. Horizontal activity, total distance traveled, movement number, stereotype count, stereotype number, and vertical movement number were significantly reduced in AAV-MethAb infected animals compared to AAV-mCherry infected animals (Fig. 3) (Each p < 0.05 for the main effect of treatment by 2-way ANOVA. For complete statistical results see Fig. 3 legend). These results demonstrate that AAV-mediated expression of the anti-Meth antibody in the periphery can reduce Meth-induced hyperactivity in mice.

### AAV-MethAb infection did not elicit immune abnormality

**Figure 4:**
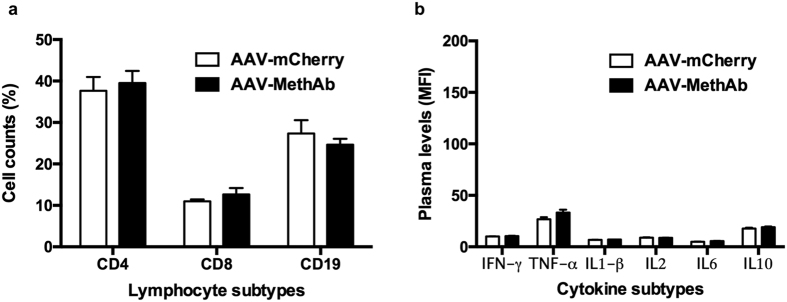
Lymphocyte counts and plasma cytokine levels in AAV-MethAb injected mice vs. those in AAV-mCherry injected mice. Mice were treated with AAV-MethAb (2.5 × 10^10^ VGC/animal; n = 8, i.p.) or AAV-mCherry (2.5 × 10^10^ VGC/animal; n = 6, i.p.). At five weeks post-infection, peripheral blood was collected for the measurement of lymphocyte counts and plasma cytokine levels. (**a**) Three main types of immune cells, including CD4+ T lymphocytes, CD8+ T lymphocytes, and CD19+ B lymphocytes, were counted by flow cytometry analysis. Data were expressed as mean percentage of total cell counts (lymphocytes + monocytes) ± SEM. (**b**) The plasma levels of six representative cytokines, including IFN- γ, TNF-α, IL1-β, IL2, IL6, and IL10, were measured by Multiplex Immunoassay using a flow cytometry-based platform (Luminex 200). Data were expressed as mean fluorescence intensity (MFI) ± SEM. No significant differences for each measured lymphocytes or cytokines were found between AAV-MethAb and AAV-mCherry injected groups.

To investigate whether the immune response following AAV infection can contribute to the difference in Meth-induced behavioral changes between mice infected with AAV-MethAb (2.5 × 10^10^ VGC/animal; i.p., n = 8) and AAV-mCherry (2.5 × 10^10^ VGC/animal; i.p., n = 6), we collected the whole blood at five weeks post-infection and subjected it to the measurement of lymphocyte counts and cytokine levels. There were no significant differences between these two groups of mice in the cell counts of three major lymphocyte subtypes, including CD4+ T lymphocytes (39.5 ± 2.9% vs. 37.6 ± 3.3%), CD8+ T lymphocytes (12.7 ± 1.5% vs. 11.0 ± 0.4%), and CD19+ B lymphocytes (24.6 ± 1.4% vs. 27.3 ± 3.2%) (Fig. 4a). The plasma levels of six representative cytokines, including four pro-inflammation cytokines (IFN-γ, TNF-α, IL1-β, IL6), one anti-inflammation cytokine (IL10), and one lymphocyte proliferation cytokine (IL2), were not significantly different between groups (Fig. 4b). When the cytokine levels were transformed from mean fluorescence intensity (MFI) into concentration (pg/ml), the levels of all cytokines were less than the lowest detected concentration (1 pg/ml) of the standard curve. These results suggest that AAV-MethAb infection did not alter any of the indices of immune status tested, compared to AAV-mCherry infection, nor induce systemic inflammation responses.

## Discussion

The purpose of the present study was to examine the effectiveness of AAV-mediated gene transfer of a Meth-specific antibody in a mouse model. A serotype-8 recombinant AAV vector (AAV-MethAb) encoding a full-length antibody with high affinity and specificity to Meth was generated by triple transfection. We demonstrated that a single dose of AAV-MethAb administered via the intraperitoneal route in mice caused Meth-specific monoclonal antibody (MethAb) production at high titers in peripheral blood for at least 29 weeks. Expression of MethAb was associated with the reduction of Meth-induced hyperactivity and Meth concentrations in the brain. We therefore conclude that AAV-MethAb administration effectively reduces Meth responses *in vivo*.

We reported a high and sustained *in vivo* expression of a functional anti-Meth monoclonal antibody following gene delivery through AAV vectors. This finding is consistent with previous reports that combination of 2A self-processing system and AAV-mediated gene transfer can achieve long-term stable expression of full-length antigen-specific antibodies in the periphery for the treatment of cancers and Alzheimer’s disease in rodent models^23^,^24^,^25^. The MethAb, generated by hybridoma cell lines, has high selectivity to Meth without cross-reactivity with a broad range of small molecules^26^. Similar high selectivity to Meth was found in the current study. We demonstrated that L-DOPA (structurally similar to Meth), bupropion, and methadone did not compete with Meth for the binding to MethAb in the sera collected from the animals receiving AAV-MethAb. These data suggest that AAV-MethAb infection produced a specific antibody for (+)-Meth *in vivo*.

The use of a selective antibody against Meth responses has been reported^6^,^11^. However, there are several disadvantages using antibody infusion. An over a 30-fold decline of serum MethAb concentration at 30 days was found after a single intravenous infusion of MethAb in rats; moreover, the infused antibody substantially lost its binding activity to Meth within 24 hours *in vivo*^27^. These findings suggest that a single anti-Meth antibody infusion may not be sufficient to neutralize Meth responses in addicts who use Meth repetitively. A well-scheduled repeated infusion of MethAb is thus required to maintain effective reductions in serum concentrations for the treatment of Meth addiction. In contrast, we found that a single dose of AAV-MethAb provided a long-term stable serum level of MethAb over 29 weeks without a loss of Meth binding activity. We therefore conclude that the newly synthesized MethAb is continuously secreted from AAV-MethAb transduced cells to maintain a stable serum level of functional MethAb. These results also suggest that AAV-mediated gene transfer may be superior to passive immunization of anti-Meth monoclonal antibodies in long-term therapy for Meth abuse.

Previous studies had demonstrated that passive immunization of purified MethAb before Meth administration increased serum total Meth levels while reducing Meth levels in the brain and other organs, suggesting that MethAb is a pharmacokinetic antagonist for Meth responses^14^,^28^,^29^. In the present study, the Meth levels in the brain and the free Meth levels in the serum, together with Meth-mediated hyperactivity, were all reduced after AAV-MethAb infection. Furthermore, these attenuation effects are not likely to be caused by an immune reaction to the presence of MethAb and viral particles, since AAV-MethAb infection did not alter immune status nor induce systemic inflammation. These data suggest that AAV-MethAb mediated expression of MethAb in the periphery can reduce Meth responses *in vivo*.

This is an initial study which demonstrates only the efficacy of the AAV-MethAb. There is no straightforward relationship between blocking the effects of Meth per se via an antibody, and the use of that antibody to treat Meth addiction. The results obtained from this work, on the other hand, pave the way for additional studies on a possible therapeutic effect of AAV-MethAb. Perhaps the next step would be the use of a self-administration model, rather than simple locomotor activity testing. Further issues may also arise; for example, it is possible that administration of AAV-MethAb to treat Meth addiction would result in the subjects abusing Meth at higher levels to overcome the blocking effect of the antibody. Additionally, partial blocking of the effects of Meth may not entirely block the rewarding effects of the drug, so that motivation to obtain Meth is increased rather than decreased. Unfortunate consequences might include greater levels of effort expended to obtain additional quantities of the drug or to obtain alternative drugs which are not blocked by MethAb. This would have the potential to lead to further toxicity while failing to treat the underlying addiction.

Meth is a highly addictive stimulant for the central nervous system and has been severely abused worldwide. There is still no effective medication for Meth addiction. In this study, we demonstrate that AAV-mediated gene transfer provides a persistent stable expression of secreted Meth-specific antibodies in the bloodstream, reduces the entry of Meth into the brain, and attenuates Meth-induced hyperactivity in mice. This gene transfer approach is potentially useful along with other therapeutic interventions for the treatment of Meth addiction.

## Methods

### Chemicals and cells

Bupropion hydrochloride (Sigma-Aldrich), L-DOPA (Sigma-Aldrich), methadone hydrochloride (U.S. Pharmacopeia), and (+)-methamphetamine (Meth; National Bureau of Controlled Drugs, Department of Health, Taiwan) were obtained as lyophilized powders. Drugs were prepared in phosphate-buffered saline (PBS) at appropriate stock concentrations (bupropion 100 mM, L-DOPA 10 mM, methadone 100 mM, Meth 100 mM), and filtered through a membrane filter with a pore size of 0.22 μm. The human embryonic kidney cell line 293 (Cat. No. 240073, Agilent Technologies), derived from the commonly used HEK293 cell line, was grown as monolayers in Dulbecco’s modified Eagle’s medium (DMEM) supplemented with heat-inactivated fetal calf bovine serum (FBS; 10%), penicillin (100 IU/ml), and streptomycin (100 μg/ml) at 37 °C in a humidified incubator with 5% CO_2_.

### Plasmid construction

The cDNA that encodes both heavy and light chains of the Meth-specific monoclonal antibody (MethAb; clone 6H4)^26^ was synthesized and cloned into the pAAV-MCS plasmid (Agilent Technologies) at EcoRI and BglII sites. The synthesized cDNA was designed to encode a fusion protein containing several peptide elements arranged in the following order: a full-length heavy chain, a furin cleavage site (RKRR), a V5 epitope (GKPIPNPLLGLDST), a spacer peptide (SGSG), a 2A self-processing sequence (APVKQTLNFDLLKLAGDVESNPGP), a full-length light chain, and a 6 × histidine tag. A post-transcriptional regulatory element derived from the woodchuck hepatitis B virus (WPRE; GeneBank accession no. J04514) was introduced at the end of the coding sequence to increase mRNA stability and protein production. The final plasmid, pAAV-MethAb, was subjected to nucleotide sequence analysis to confirm the fidelity of the inserted cDNA.

### Immunocytochemistry

At 24 h post-transduction with AAV-MethAb, 293 cells grown on 12-mm glass coverslips were washed twice with PBS, fixed with 4% formaldehyde in PBS for 10 min and then permeabilized with 0.3% Triton X-100 in PBS for 10 min. The cells were incubated with 4% bovine serum albumin (BSA) in PBS to block nonspecific binding of the antibodies and then incubated with an Alexa-Fluor-488-conjugated anti-mouse-IgG goat polyclonal antibody (1:1,000, Invitrogen) for 30 min, followed by washing with PBS three times for 5 min each. The cells were fixed again with 4% formaldehyde for 10 min and washed with distilled water once. The coverslips were then mounted on glass slides by using a mounting medium (Vector Laboratories), which contains 4′, 6-diamidino-2-phenylindole (DAPI; 1.5 μg/ml) for localizing the nucleus. Cells were then examined, and fluorescence images were obtained by using an Axiovert 200 M microscope system (Carl Zeiss).

### Western blotting

Western blot analysis was performed at room temperature as reported previously^30^. For the denatured-reducing condition, the purified MethAb, anti-NeuN mouse monoclonal antibody (Cat. No. MAB377, Millipore), or purification control sample was mixed with an equal volume of 2 × Laemmli buffer (containing 4% SDS, 125 mM Tris-HCl pH6.8, 10% β-mercaptoethanol, 20% glycerol, and 0.004% bromophenol blue in distilled water). For the denatured-nonreducing condition, β-mercaptoethanol was not added in the 2 × Laemmli buffer. All sample mixtures were heated at 90 °C for 10 min and then separated by sodium dodecyl sulphate-polyacrylamide gel electrophoresis (SDS-PAGE) on 12% gels. The separated proteins were electrophoretically transferred from the gel to a PVDF membrane (Cat. No. RPN303F, GE Healthcare). The membrane was incubated with blocking buffer [5% skim milk (Cat. No. 70166, Sigma-Aldrich, St Louis, MO, USA) and 0.1% Tween-20 in PBS] for one h with gentle agitation. The membrane was then incubated with a horseradish peroxidase (HRP)-conjugated anti-mouse-IgG goat polyclonal antibody (1:2000; Cat. No. GTX213111-01, GeneTex) or an HRP-conjugated anti-6 × His mouse monoclonal antibody (1:2000; Cat. No. R931-25, Invitrogen) in 10 ml blocking buffer for 2 h with gentle agitation, followed by washing with 0.1% Tween-20 (in PBS) three times for 10 min each. The light emission signal of the target proteins on the PVDF membrane was generated by using an enhanced chemiluminescence reagent (Cat. no. RPN2106, GE Healthcare) and then detected by an X-ray film (Cat. No. NEF596, Kodak).

### Virus production and titration

The recombinant adeno-associated virus serotype-8 vector (AAV-MethAb) was produced by triple plasmid transfection without the use of a helper virus. The 293 cells were seeded at 2 × 10^6^ per 10-cm culture plate in 10 ml of DMEM plus 10% FBS for 48 h before transfection. Subconfluent (70–80%) monolayer cells were co-transfected with pAAV-MethAb (10 μg), p5e18-VD2/8 (10 μg; Cat. No. P0007, University of Pennsylvania)^31^, and pHelper (10 μg; Agilent Technologies) by calcium phosphate precipitation (Cat. No. 631312, Clontech). The plasmids used for transfection were purified from *E. coli* DH5α by the anion-exchange-based endotoxin-free plasmid purification kit (Cat. No. 12362, Qiagen). At six h post-transfection, the culture medium was changed with fresh DMEM plus 2% FBS, and the cultures were further incubated for 48 h. The culture media were collected and centrifuged at 2,500 × g at 4 °C for 30 min to remove the cell debris. The supernatants were filtered through a 0.22 μm filter, mixed with 40% PEG8000 (in 0.15 M NaCl) to a final concentration of 8%, and incubated at 4 °C overnight. The mixture was centrifuged at 2,500 × g at 4 °C for 1 hour, and the obtained pellet was dissolved in PBS in 1/80 of the starting volume. The viral suspension was aliquoted and stored at −80 °C until use.

Viral titers were determined by the quantitative real-time PCR assay (qPCR)^32^ performed on an ABI StepOnePlus system. Five microliters of the viral sample were pre-treated with two units of DNase I (Cat. No. M0303, New England BioLabs) in a final volume of 50 μl at 37 °C for 1 hour, and then DNase I was inactivated at 75 °C for 10 min. The primers that target WPRE on the vector plasmid (pAAV-MethAb) to amplify a 384 bp fragment of the qPCR product were designed by the Primer-3 program and listed as follows: 5′-TCATGCTATTGCTTCCCGTATGG-3′ (forward), 5′-GGATTGAGGGCCGAAGGGA-3′ (backward). Each reaction mixture (20 μl) contained 2 μl of DNase-pretreated viral sample, 10 μl of 2 × SYBR green PCR master mix (Cat. No. 4367659, ABI), and 0.5 μM of each primer. The PCR cycling program was set as the following: 95 °C for 10 min followed by 40 cycles for amplification (95 °C for 15 sec, and 60 °C for 30 sec), and a cycle for generating a melting curve (95 °C for 15 sec, 60 °C for 1 min, and 95 °C for 15 sec). A standard curve using a ten-fold serial dilution (0.01–100 pg) of the vector plasmid (pAAV-MethAb) was generated in every qPCR assay. Viral titers were expressed as viral genome copies per milliliter of the virus sample (VGC/ml).

### Colorimetric assay

293 cells, seeded in 6-well plates at a density of 5 × 10^5^ cells/well overnight, were transduced with AAV-MethAb at a multiplicity of infection (MOI) of 10^2^ VGC/cells. At 48 h post-transduction, the culture medium samples were collected for the purification of MethAb. As a purification control, a commercially available mouse monoclonal anti-V5 antibody (10 μg; Cat. No. GTX42525, GeneTex) was added into the culture medium collected from non-transduced cells. Each collected medium sample (2 ml) was mixed with 10 μl of 10% (v/v) Protein-G Magnetic Beads (Cat. No. 28-9670-66, GE Healthcare) with thorough agitation for two h at 4 °C to purify the antibodies, followed by washing the beads with PBS six times. The purified antibodies, remaining attached to the beads, were incubated with 50 μl of HRP-conjugated (+)-Meth with thorough agitation for two h at 4 °C. After washing six times with PBS to remove unbound (+)-Meth-HRP, the TMB chromogenic substrate (100 μl) was added and incubated for 15 min. The reaction of color development was terminated by adding 1 N hydrochloride (100 μl), and the absorbance was measured at a wavelength of 450 nm.

### Animals and injection with AAV-MethAb viral vectors

Male ICR mice were purchased (BioLASCO Co., Ltd. Taiwan) at 7 weeks of age and group-housed under environmentally controlled conditions for one week before the beginning of experimental procedures. Mice were intraperitoneally injected with 500 μl of PBS, AAV-MethAb (10^9^ or 2.5 × 10^10^ VGC/animal), or AAV-mCherry (2.5 × 10^10^ VGC/animal). Blood samples were collected from tail veins at various time points (before injection, and 2 to 29 weeks after injection), allowed to clot at room temperature for 30 min, and centrifuged at 10,000 × g for 10 min. Sera were collected from the upper layer and stored at −80 °C until use for the measurement of antibody levels. This study was carried out in strict accordance with the recommendations in the Guide for the Care and Use of Laboratory Animals of the National Institutes of Health. The animal protocol was reviewed and approved by the Animal Research Ethics Board at National Health Research Institutes in Taiwan (Permit Number: NHRI-IACUCC-104035-A). While conducting animal experiments, all efforts were made to minimize any suffering.

### Locomotor activity

At five weeks after injection with AAV-MethAb (2.5 × 10^10^ VGC/animal; i.p., n = 8) or AAV-mCherry (2.5 × 10^10^ VGC/animal; i.p., n = 6), each mouse was placed individually into a transparent acrylic chamber (42 × 42 × 30 cm; length × width × height), equipped with horizontal and vertical infrared sensors (AccuScan Instruments, Inc.). After a 30-min habituation, animals were intraperitoneally injected with a dose of Meth (1 mg/kg), and the parameters of six locomotor activities were recorded for 2 h. Total distance traveled (TOTDIST): the distance in centimeters traveled by the animals. Horizontal activity (HACTV): the number of bean interruptions detected by the horizontal sensor. Movement number (MOVNO): the number of horizontal movements followed by a break that occurs for a period greater than one second. Stereotype count (STRCNT): the number of bean interruptions during the period of stereotypic episodes. Stereotype number (STRNO): the number of stereotypic behavior that is followed by a break with a period equal or greater than one second. Vertical movement number (VMOVNO): the number of vertical movements followed by a period during which the animal must go below the level of the vertical sensor for at least one second.

### Antibody titration

Serum sample (20 μl), collected from mice receiving AAV-MethAb or PBS, were purified by 5 μl of 10% (v/v) Protein-G Magnetic Beads. The purified antibodies, remaining attached to the beads, were incubated with 50 μl of (+)-Meth-HRP with thorough agitation for 2 h. After washing with PBS six times to remove unbound (+)-Meth-HRP, the TMB chromogenic substrate (100 μl) was added and incubated for 15 min. The color development was terminated by adding 1 N hydrochloride (100 μl), and the absorbance was measured at a wavelength of 450 nm. The Meth-specific antibody titer was calculated by the formula, 50 × (O.D.450 value of the sample), and expressed as unit per milliliter of serum (U/ml).

### Measurements of methamphetamine levels

Mice were sacrificed 10 minutes after Meth injection (1 mg/kg, i.p.). The brain tissue, homogenized in PBS, and serum were collected and stored at −80 °C until use. The Meth levels in the samples were measured by using the Meth Direct ELISA kit (Cat. No. KA0934 V0.2, Abnova), based on the competition of HRP-labeled Meth binding to the Meth-specific antibodies. Before the ELISA assay, each serum sample (20 μl) was incubated with 10 μl of 10% (v/v) Protein-G Magnetic Beads to deplete antibody-bound Meth. The Meth levels in the brain and serum were expressed as nanograms per gram of brain (ng/g), and nanograms per milliliter of serum (ng/ml), respectively.

### Binding capacity of anti-Meth antibody

293 cells, seeded in 10-cm dishes at a density of 2.5 × 10^6^ cells/dish overnight, were transfected with or without pAAV-MethAb (8 μg for each dish) by jetPEI transfection reagent (Cat. No. 101-40 N, PolyPlus), as per manufacturer’s instructions. At 48 h post-transfection, the culture medium was subjected to antibody purification. Briefly, each collected medium sample (10 ml) was mixed with 100 μl of 5% (v/v) His-Mag Sepharose beads (Cat. No. 28-9673-88, GE Healthcare) with thorough agitation for 2 h at 4 °C, followed by washing the beads with PBS six times. The purified antibodies, remaining attached to the beads, were drained and then incubated with 150 μl of Meth (50 ng/ml) with thorough agitation for 2 h at 4 °C to pull down Meth molecules. After that, beads were collected using a magnet, and the supernatant was subjected to the quantification of Meth, as described previously. The precipitated beads and a quantified anti-NeuN mouse monoclonal antibody were subjected to Western blot analysis to quantify the purified MethAb. The heavy and light chains of the antibodies were detected by using an HRP-conjugated anti-mouse-IgG goat polyclonal antibody (Cat. No. 115035003, Jackson Lab). The ImageJ software was used to integrate the image density of detected antibody fragments. The MethAb concentrations were estimated through a standard curve (y = 1.905 × 10^−6x^ + 0.0111, R^2^ = 0.9895) which was created by plotting the image intensity of anti-NeuN as a function of mass. The Meth binding capacity of MethAb was calculated by the following formula, {[(the amount of Meth pulled down by His-Mag Sepharose beads + MethAb)–(the amount of Meth pulled down by His-Mag Sepharose beads)]/(the molecular weight of Meth)}/[(the mass of purified MethAb used to pull down Meth molecules)/(the molecular weight of MethAb)], and expressed as numbers of Meth molecule per molecule of MethAb.

### Competition binding assay

Sera were incubated with (+)-Meth-HRP in the presence of different competitors (bupropion, methadone, Meth, or L-DOPA) at room temperature for 2 h. Antibodies in each binding reaction were purified by 5 μl of 10% (v/v) Protein-G Magnetic beads and then incubated with 50 μl of TMB chromogenic substrate for 30 min. After adding 50 μl of 1 N hydrochloride to stop the reaction, samples were read at 450 nm. The intensity of the color developed was inversely proportional to the inhibition of binding to (+)-Meth-HRP.

### Measurements of lymphocytes and cytokine levels

Approximately 200 μl of whole blood was collected from each mouse by tail-tip transection into EDAT-containing tubes (Cat. No. 450474, Greiner Bio-One,). The blood samples were immediately mixed well by inverting the tube five times to prevent clotting. After centrifugation at 3,000 rpm at room temperature for 15 min, the plasma in the upper layer was subjected to cytokine quantification for IFN-γ, TNF-α, IL1-β, IL2, IL6, and IL10, using ProcartPlex Multiplex Immunoassays (Affymetrix) and a flow cytometric platform (Luminex 200). The data of cytokine levels were analyzed by ProcartlPlex software and reported as mean fluorescence intensity (MFI) ± standard error (SEM). The rest part of each blood sample was mixed with 5 ml of 1 × ACK buffer (150 mM NH_4_Cl, 10 mM KHCO_3_, 1 mM EDTA) on ice for 5 min to lyse erythrocytes and then centrifuged at 1,000 rpm at 4 °C for 5 min to precipitate immune cells. This erythrocyte lysing procedure was conducted twice. After that, the cell pellet was resuspended in 600 μl of FC buffer (1 × PBS containing 1% FBS) and evenly divided into three portions. Each portion of aliquots was incubated with fluorophore-labeled CD4-APC (1:250, Cat. No. 100411, BioLegend), CD8-PE (1:250, Cat. No. 100707, BioLegend), or CD19-APC (1:250, Cat. No. 152409, BioLegend) rat monoclonal antibody on ice for 1 h. After wash once with FC buffer, each cell pellet was resuspended in 500 μl of FC buffer and analyzed by a FACSCalibur flow cytometer (BD Biosciences) using FlowJo software 7.6. A total of 10^5^ events were acquired. The lymphocyte/monocyte populations were gated based on their forward scatter (FSC) and side scatter (SSC) properties and further analyzed for their expression of CD4, CD8, and CD19 cell markers. Data were expressed as mean percentage of total cell counts (lymphocyte + monocyte) ± SEM.

### Statistical analysis

Data were expressed as means ± standard error (SEM) and analyzed by using GraphPad Prism 6.0 statistical program (GraphPad Software, Inc.). The assumption of data normality was checked by D’Agostino & Pearson omnibus normality test, and no violation of assumption was found. Statistically significant difference was considered when the two-tailed p-value was less than 0.05. Student’s *t*-test was applied to assess the difference between two groups. Comparisons for Meth-induced behavioral changes or MethAb expression levels between groups at different time points were performed by two-way repeated-measures analysis of variance (ANOVA), with post-hoc analysis by Sidak’s multiple comparison test for Fig. 2a.

## Additional Information

**How to cite this article:** Chen, Y.-H. *et al*. Recombinant Adeno-Associated Virus-Mediated Expression of Methamphetamine Antibody Attenuates Methamphetamine-Induced Hyperactivity in Mice. *Sci. Rep.* **7**, 46301; doi: 10.1038/srep46301 (2017).

**Publisher's note:** Springer Nature remains neutral with regard to jurisdictional claims in published maps and institutional affiliations.
